# Social isolation among adolescents and its association with depression symptoms

**DOI:** 10.1186/s43045-023-00314-4

**Published:** 2023-05-05

**Authors:** Danah A. Alsadoun, Haneen S. Alotaibi, Amwaj I. Alanazi, Leena A. Almohsen, Njoud N. Almarhoum, Samira Mahboub

**Affiliations:** Department of Health Sciences, College of Health and Rehabilitation Sciences, Princess Nourah Bint Abdulrahman University, P.O. Box 84428, Riyadh, 11671 Kingdom of Saudi Arabia

**Keywords:** Social isolation, Depression, Socialization, Adolescents

## Abstract

**Background:**

Social isolation can be defined as the absence of social interactions, contacts, and relationships with family, friends, and neighbors. According to some studies, social isolation was associated with depressive symptoms. At least one out of every five teenagers suffer from a diagnosable mental health problem that impairs their function. Saudi Arabia has a significant adolescent population; however, there are few studies conducted in Saudi Arabia to assess social isolation and its psychological impact among adolescents. This research is intended to study the prevalence of social isolation among adolescents in Riyadh and its association with depression symptoms.

**Methods:**

A cross-sectional study was conducted among 483 adolescents living in Riyadh, Saudi Arabia. An online self-administered questionnaire was used, and it was composed of three sections which are sociodemographic characteristics, assessment of social isolation, and assessment of depression symptoms.

**Results:**

The study reported the prevalence of social isolation among adolescents was 10.14%. The prevalence of depression symptoms among adolescents was high (31.68%). There was a significant association between social isolation and symptoms of depression among the studied sample (*χ*^2^ = 12.3, *p* = 0.002). It was found that being a male, living with both parents, and having low income are significant predictors of social isolation among adolescents; with *r*^2^ = 0.08 and *p*-value < .0001. It was also found that low-income level had a more impact on social isolation among adolescents than other factors (estimate = 1.6).

**Conclusions:**

A total of 10% of adolescents in Riyadh are socially isolated. There is a significant association between social isolation and symptoms of depression among the studied adolescents. Symptoms of depression can be predicted by social isolation.

**Recommendations:**

The Ministry of Health and the Ministry of Education in Saudi Arabia should work together to conduct educational intervention programs for adolescents about mental health, the importance of social interaction, and the drawbacks of social isolation. These topics should also be involved in the curriculum of secondary schools.

## Background

Social isolation can be defined as the absence of social interactions, contacts, and relationships with family, friends, and neighbors [1]. Some factors may lead to social isolation such as physical disabilities. Some people feel ashamed about their disability and consequently limit their social contact. Other factors that may lead to social isolation include being geographically separated from family and friends and the misuse of social media [2]. Moreover, physical distancing and other preventive measures during COVID-19 pandemic can also lead to social isolation [2]. Lastly, the small number of family members, and the weak relationship with neighbors and relatives, can lead to social isolation [3].

Between ages of 18 and 79 years, 12.3% of people suffer from being socially isolated [4]. Older persons are more subjected to social isolation because they are operationally very dependent on family members or community services [5]. Due to COVID-19 and its precautionary measures including limitation of close social contact, adolescent mental health may be affected, and they are more prone to experience high rates of depression and anxiety [6].

According to some studies, social isolation was associated with depressive symptoms [6]. Depression is a common and dangerous condition in adolescence. It is a disease that affects a person’s emotions, sleep, energy, appetite, and attention [7]. Depression is the fourth most common cause of disease and impairment in teenagers aged 15 to 19 [8].

Adolescence is the era of transition between childhood and adulthood. It entails significant physical changes. Both adolescents and their families may experience excitement and anxiety due to the numerous physical, sexual, cognitive, social, and emotional changes that occur during this period. Adolescence is divided into three stages: early adolescence (10–13 years old), middle adolescence (14–17 years old), and late adolescence (18–21 years old). Late adolescents have reached the full adult height and have completed their physical development. By this time, they should have more impulse control and accurately assess risks and rewards [9]. At least one out of every five teenagers suffer from a diagnosable mental health problem that impairs their function [10]. Saudi Arabia has a significant adolescent population [11]. In Riyadh, the age groups (15–19 years) are 538,455 [12]. There are a few studies conducted in Saudi Arabia to assess social isolation and its psychological impact among adolescents in Riyadh. Adolescents are a susceptible group because they go through considerable changes, as well as a biological phenomenon that produces hormone shifts [11, 13]. Adolescent mental health disorders are a big problem, but they are also quite frequent and treatable [10]. Therefore, this research is intended to study the prevalence of social isolation among adolescents in Riyadh and its association with depression symptoms.

## Subjects and methods

### Study design and sitting

A cross-sectional study was conducted in Riyadh.

### Study population

They are adolescents who are in the age of 18 to 21 years of both genders and living in Riyadh.

### Exultation criteria

‏Adolescents diagnosed with autism or attention-deficit hyperactivity disorder (ADHD) were excluded from the study.

### Study duration

Data collection was between January and March 2022.

### Sample size

The sample size was calculated by using n4Studies software. The study population is more than 10,000, so the sample was calculated using a 95% confidence interval, 0.5 proportion, 0.05 degree of accuracy, and 80% power of the study. The calculated sample from the equation: $n=\frac{{\mathrm{Z}}^{2}\mathrm{P}(1-\mathrm{P})}{{\mathrm{d}}^{2}}$ was 385. The total participants eventually included in the study were 483.

### Sampling technique

The questionnaire was designed and conducted online using the convenience sample technique. The questionnaire link was sent to people from different social media outlets such as WhatsApp and Twitter.

### Data collection tools

An online questionnaire was used, and it is composed of three sections which are sociodemographic characteristics, assessment of social isolation, and depression symptoms.

The first section included sociodemographic questions such as nationality, age, gender, education level, income, and residence.

The second section included assessment of social isolation using a modified scale [14]. It contained 7 questions about social contact during the last month and one question about number of friends.

Questions assessing social contact included the following:

- How often did you make contact with your family members or relatives who are living apart by voice call or video call?
- How often did you make contact with your friends or neighbors by email or text message?
- How many times did you go out to see a friend?

The responses were presented on a 7-point Likert scale (6–7 times a week, 4–5 times a week, 2–3 times a week, once a month, and never). The score was calculated to get the number of contacts per month for each question. The number of weeks in a month was calculated by 365 days in a year/12 months in a year/7 days in a week = 4.35 weeks in one month. Such as if a participant selected 6–7 times a week, the mean of 7.6 times in a week is (7 + 6/2) = 6.5, so the score is equal to (6.5 [days in a week] × 4.35 [weeks in a month] = 28.28 times per month. If a participant selected 4–5 times a week, the score will be 19.59 times per month, 2–3 times a week equals 10.88 times per month, once a week equal 4.35 times per month, 2–3 times a month equals 2.5 times per month and once a month equal 1 time per month, and never equal 0. In addition to one more question about the number of friends, “How many classmates can you communicate with about study issues” with 5 responses which were (7 or more, 5–6 colleagues, 3–4 colleagues, 1–2 colleagues and none). Those who have one or more colleagues had a score of 1, and those who select “none” have a score of zero.

The sum of all 7 questions of social contact responses was calculated. The equation used to categorize participants as being socially isolated or not is as follows: total contact < 4.35 times per month or number of friends equals 0. The participants were classified into two categories: participants who had total contact < 4.35 per month or a score of 0 in the number of friends were considered socially isolated, and the participants who had total contact > 4.35 per month or a score of 1 in the number of friends were socially integrated.

The last section included nine items, to assess symptoms of depression using a modified Adolescent Depression Rating Scale (ADRs) [15]. It included 9 statements such as the following: I have no energy for work/school, I have trouble thinking, I feel overwhelmed by sadness and listlessness, nothing really interests or entertains me, what I do is useless, everything annoys me, I feel downhearted and discouraged, I sleep badly, school/work doesn't interest me just now, and I can't cope, with 5-point Likert scale responses (always, usually, sometimes, rarely, never). Always response scored 5 points, and never scored 1 point. The total depression symptoms score was calculated by using the formula as follows: the sum of all 9 question responses. The maximum score is 45, and the minimum is 9. Participants were categorized into negative, neutral, and positive. It is considered negative from 9 to 21, neutral from higher than 21 to 33, and positive if higher than 33.

### Validity and reliability

#### Cross-translation validity

To ensure the validity of the translation, a cross-translation was done by translating the original English version of the questionnaire to Arabic, and this was sent to an expert, who translated the questionnaire again from Arabic to English language. Then, both English versions were compared to each other to ensure the accuracy of the translation.

#### Face validity

Face validity was done by sending both questionnaires of social isolation and depression symptoms to an associate professor of clinical psychology at the Princess Nourah University. Few modifications were done to ensure the validity of the questionnaire according to the given feedback.

Validity was tested statistically for each question separately. All questions were significantly positively correlated with the total score, *r* was more than 0.3 for all questions, and *p*-values were all less than 0.05.

#### Reliability

The reliability of both questionnaires of social isolation and depression symptoms was tested separately using Cronbach’s alpha by JMP. For social isolation questionnaire, *α* was 0.7, and for the depression symptoms questionnaire, *α* was 0.9.

### Statistical analysis

Statistical analysis was done using John’s Macintosh Project (JMP) version 14 pro. Data were managed by tabulation according to their level of measurement. Numerical variables were presented using median and interquartile range (social contacts) because the data was not normally distributed, and categorical variables were presented in frequency tables. The chi-square test was used to assess the association between social isolation and depression symptoms. To find out if social isolation is a predictor for depressive symptoms and to minimize the effect of confounding factors, multiple linear regression model was used to predict score of depression symptoms using social isolation and different demographic criteria. Logistic regression was used to predict social isolation based on sociodemographic characteristics. The cutoff point for significance was 0.05.

## Results

Table 1 demonstrates sociodemographic characteristics of the sample. A total of 79.92% of them were females, and about 69.75% of the samples were college students. Regarding residency, almost half of the participants lived in the east, about 66.25% of the participants reported having enough income, while 7.66% of them had low income. More than half of them lived in small families; also, the majority of them were living with their parents (87%).

**Table 1:** Sociodemographic characteristics of the studied sample (*N* = 483)

**Variable**	*N*	%
**Gender**		
Females	386	79.92%
Males	97	20.08%
**Educational level**		
High school and less	147	30.43%
University	336	69.57%
**Residency**		
South of Riyadh	64	13.25%
East of Riyadh	171	35.40%
North of Riyadh	162	33.54%
West of Riyadh	52	10.77%
Center of Riyadh	34	7.04%
**Family income**		
Not enough	37	7.66%
Enough	320	66.25%
Enough and saving	126	26.09%
**Number of family members**		
Large family (more than 7)	190	39.34%
Small family (7 or less)	293	60.66%
**Living condition**		
With both parents	422	87.37%
With only one parent or someone else	61	12.63%

Table 2 demonstrates a description of social activities during the last month among adolescents. It shows that the highest average was for “How many times did you communicate with your friends/neighbors by text messages” (19.59 ± 23.9), while the lowest was for “How many times did you communicate with your family members or relatives who are living apart by voice/video call,” “going out to meet a friend,” and “Number of attended social events” that were equal to 2.5 for each.

**Table 2:** Descriptive statistics of social contact during the last month among adolescents

Items	Median	Interquartile range
How many times did you communicate with your family members or relatives who are living apart by text messages?	10.88	17.09
How many times did you communicate with your family members or relatives who are living apart by voice or video call?	2.5	9.88
How many times did you communicate with your friends or neighbors by text messages?	19.59	23.93
How many times did you communicate with your friends or neighbors by voice or video call?	4.35	18.59
How many times did you go out to meet a friend?	2.5	9.88
Number of social events you attended	2.5	3.35
Number of received phone calls	10.88	17.09

Table 3 represents the prevalence of social isolation and symptoms of depression among adolescents. A total of 10.1% of the participants were socially isolated, while 89.9% were not. Also, the distribution of depression symptoms revealed that 25% were negative, 42.8% were neutral, and 31.6% were positive.

**Table 3:** Prevalence of social isolation and symptoms of depression among adolescents

Socially isolated	*N*	%
No	434	89.86%
Yes	49	10.14%
**Depression symptoms categories**		
Negative	123	25.47%
Neutral	207	42.86%
Positive	153	31.68%

Table 4 represents the association between social isolation and depression symptoms among adolescents. It was found that 53.06% of socially isolated adolescents had positive depression symptoms compared to 29% of those who are not. This association is statistically highly significant (*χ*^2^ = 12.3, *p* = 0.0021).

**Table 4:** The association between social isolation and symptoms of depression among adolescents

Depression symptoms Categories	Socially isolated		Chi-square test
	**No**	**Yes**	
**Negative**	112 25.81%	11 22.45%	*χ*^2^ = 12.36**
**Neutral**	195 44.93%	12 24.49%	
**Positive**	127 29.26%	26 53.06%	

^**^p-value < 0.01

Further data analysis was conducted to find out the predictors of depression symptoms with multiple linear regression model as demonstrated in Table 5. The regression model was used to find out if the association between social isolation and depression symptoms was due to the presence of confounding factors or not. The dependent variable in the model was the score of depression symptoms, and the independent variables were as follows: social isolation and different sociodemographic factors (gender, age, educational level, income, family size, and living with both parents or not). Only income and social isolation were the significant predictors for depression symptoms (*p*-value of the whole model 0.001, *r*^2^ = 0.04).

**Table 5:** Multiple linear regression model to determine predictors of symptoms of depression among the studied sample

Term	Estimate	Std. error	*t*-ratio	Prob. >|*t*|
Intercept	33.8	2.2	15.15	< .0001*
Gender (female)	0.8	0.5	1.63	0.1048
Age (young adolescent-old adolescent)	− 0.03	0.8	− 0.04	0.9658
Educational level (college-high school)	0.56	0.9	0.61	0.5389
Income (satisfied-not satisfied)	− 4.7	1.6	− 2.93	0.0036*
Family size	0.01	0.1	0.07	0.9416
Living with both parents (other)	0.6	0.6	0.96	0.3389
Socially isolated (no)	− 1.9	0.7	− 2.73	0.0066*

^*^p-value < 0.05

Table 6 represents logistic regression model that predicts social isolation based on sociodemographic characteristics. The dependent variable is social isolation, and the independent variables entered in the model were: gender, income level, family size, and living with both parents or not. It was found that being a male, living with both parents, and having low income are significant predictors of social isolation, with *r*-square = 0.08 and *p*-value < 0.0001. It was also found that low-income level had a more impact on social isolation among adolescents than other factors (estimate = 1.6).

**Table 6:** Logistic regression model predicting social isolation based on sociodemographic characteristics

	**Estimate**	**Std. error**	**Chi-square**	**Prob. > chi-sq**
Intercept	− 1.1	0.4	5.5	0.01*
Gender (female)	− 0.5	0.1	9.3	0.002*
Income (satisfied-not satisfied)	− 1.6	0.4	14.5	0.0001*
Live with both parents (no)	− 0.8	0.3	4.5	0.03*
Family size (large families)	0.1	0.1	0.4	0.4

^*^p-value < 0.05. For log odds of yes/no

## Discussion

This study aimed to evaluate the level of social isolation among adolescents in Riyadh, in addition to assess the association between social isolation and depression symptoms among adolescents in Riyadh.

Regarding social isolation among adolescents, 10% of participants in this study suffered from social isolation. This could be due to the data were collected during COVID-19 pandemic in Saudi Arabia (from December 2021 to March 2022). A similar result was reported from a study conducted in Osaka that stated the prevalence of social isolation after the pandemic was 27.9% [14]. During COVID-19 pandemic, the preventive measures included lockdown and social distancing. These measures had a significant impact on social isolation [14].

This study showed that one-third of adolescents suffered from depression symptoms, while in another study conducted in China during the pandemic, less prevalence of depression was reported among children and adolescents (19.7%) [16]. This discrepancy could be due to younger age group involved in the study of China, where the mean age was 11 years ± 2, while in the present study it was 19 years ± 1.

The current study demonstrated a significant association between social isolation and depression symptoms among adolescents in Riyadh. It was also found that social isolation was a significant predictor of depression symptoms. Lack of social interaction can adversely affect mental health. This is consistent with another study that reported a significant positive correlation between social isolation score and depression score, which *p*-value 0.01 and *r* = 0.2381 [17]. These findings were similar to a study conducted in Denmark, showing that depression has increased due to social isolation [18]. This could be due to the social isolation resulting from COVID-19 preventing people from going out and communicating with family and friends. This causes difficulties, especially for adolescent students, and impacts mental health.

This study showed that those who have inadequate income were socially isolated. This could be because low income among adolescents is associated with financial difficulties that hinder their access to entertainment and spending time with their friends. Similarly, a previous study conducted in Canada showed that participants with a low income had experienced higher social isolation [19]. This was attributed in that later study to the fact that adolescents may mix with people less as a result of the low family income.

In this study, the participants who lived with their parents were more socially isolated than those who lived with only one parent or with other relatives. The reason behind that could be their reliance on their parents or the parents’ assistance in life demands.

The current study demonstrates that there is a significant association between social isolation and gender; it found that males are more socially isolated than females. It can be attributed to the sample being adolescents who are single, and according to previous studies of gender difference in social isolation, the never married male is more likely to be isolated than a single female because they do not create adequate emotional intimacy when they are not in partnership with a significant other [20, 21].

## Conclusions

In conclusion, it was found that 10% of adolescents in Riyadh were socially isolated. There were high levels of depression symptoms (31.68%) among adolescents. Social isolation has a significant association with depression symptoms and can be used to predict them. It was found that social isolation can be significantly predicted by gender, income, and living with both parents. It was also found that low-income level had a more impact on social isolation among adolescents than other factors.

### Recommendations

It is recommended to conduct further studies about social isolation and its association with depressive symptoms in adolescents of different stages (early, middle, and late adolescents).

The Ministry of Health and the Ministry of Education should work together to conduct educational intervention programs for adolescents about the importance of social interaction and the drawbacks of social isolation. These topics should be also involved in the curriculum of secondary schools. It is also recommended for secondary schools to have a tool to identify students suffering from social isolation and help them engage in different activities with other students at school.

## Data Availability

The datasets used are available from the corresponding author on reasonable request.
